# Delayed epidural pseudoaneurysm following cervical laminectomy and instrumentation in a patient with canal stenosis secondary to skeletal fluorosis: A case report

**DOI:** 10.1097/MD.0000000000009883

**Published:** 2018-02-23

**Authors:** Yinze Diao, Yu Sun, Shaobo Wang, Fengshan Zhang, Shengfa Pan, Zhongjun Liu

**Affiliations:** Department of Orthopaedics, Peking University Third Hospital, Beijing, China. Beijing Key Laboratory of Spinal Disease, Beijing, China.

**Keywords:** cervical spine surgery, late-onset bleeding, pseudoaneurysm, skeletal fluorosis, vertebral artery injury

## Abstract

**Rationale:**

The typical intraoperative presentation of vertebral artery injury (VAI) usually involves profuse bleeding and requires immediate treatment. However, an occult VAI may occur intraoperatively and result in delayed life-threatening epidural pseudoaneurysm several days postoperatively.

**Patient concerns:**

A 21-year-old man with compressive cervical myelopathy resulting from canal stenosis of skeletal fluorosis underwent decompression of C1 to C7 and instrumentation from C2 to C7. No impressive bleeding event occurred during the operation. On postoperative day 40, progressive quadriplegia developed.

**Diagnoses:**

Pseudoaneurysm of the VA was established by angiography.

**Interventions:**

After occlusion of the right VA, the patient underwent hematoma clearing.

**Outcomes:**

Fortunately, the patient experienced significant recovery of neurologic function after the second surgery.

**Lessons:**

From this case, we realize even in the absence of obvious signs of VAI during a cervical operation, postoperative evaluation should be mandatory for suspected bleeding events occurring at VAI-prone sites during surgery. Moreover, the bone morphological abnormality of skeletal fluorosis was determined to be the most important risk contributing to VAI in this case. The safety limits of bone removal should be determined preoperatively to avoid the effects of bone morphological abnormalities.

## 1 Introduction

Iatrogenic vertebral artery injury (VAI) is a rare but catastrophic complication of cervical or craniocervical surgery, with an overall incidence between 0.14%^[1]^ and 0.2%^[2]^ in large series. Usually, the typical intraoperative presentation of VAI is sudden and profuse bleeding that is difficult to control. However, in the current case, VAI during operation presented as a mild hemorrhage and was easily controlled by compression with pieces of gelatin sponge, similar to venous plexus rupture in the upper cervical region. This led to the neglect of possible VAI by the surgical team and resulted in the development of a life-threatening late-onset pseudoaneurysm. Hypertrophy of bone and cervical canal stenosis secondary to skeletal fluorosis contributed to the injury, and this possibility has not been thoroughly discussed in the literature.

## 2 Case report

The study was approved by the Institutional Review Board and Ethics Committee of Peking University Third Hospital. Informed consent was obtained from the individual participant included in the study.

**Figure 1:**
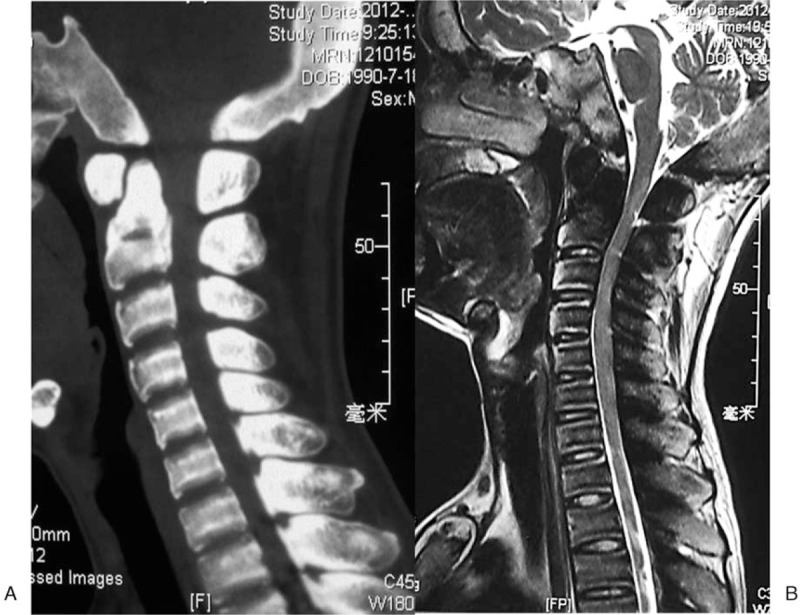
Preoperative CT and MRI of the cervical spine. (A) Preoperative CT demonstrated significant hypertrophy of bone and canal stenosis. (B) Preoperative MRI demonstrated cervical cord compression.

A 21-year-old male from a known high fluorine area presented progressing neurologic dysfunction. Imaging examinations demonstrated bone hypertrophy and canal stenosis (Fig. 1). The patient underwent posterior decompression with resection of the posterior arch of atlas as well as laminectomy from C2 to C7. Pars screws were applied on C2. Lateral mass instrumentation was performed from C3 to C7. No severe bleeding occurred during the operation, and the estimated blood loss was 500 mL. The mJOA score recovered from 4.5 points preoperatively to 13 at 4 weeks postoperatively.

**Figure 2:**
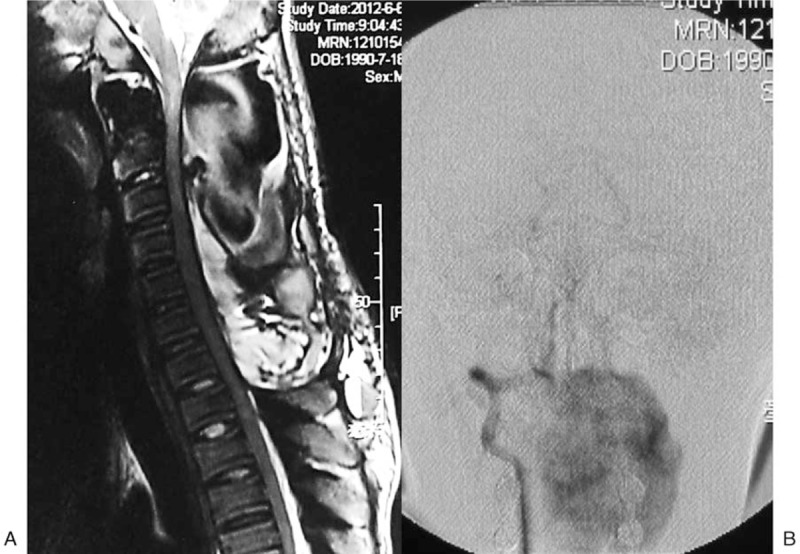
Cervical MRI and angiography on postoperative day 40. (A) MRI examination demonstrated a large epidural hematoma and extensive spinal cord compression. (B) The bleeding point was located at the right V3 segment in the VA groove of the atlas.

On postoperative day 40, however, the patient developed progressive quadriparesis within 4 hours. A cervical MR examination demonstrated a large epidural hematoma and spinal cord compression (Fig. 2A). A bedside ultrasonography showing swirl-like arterial blood flow signals in the hematoma indicated a pseudoaneurysm from the right VA. Angiography showed bleeding of the right V3 segment in the vertebral artery groove of the atlas (Fig. 2B). The right VA was occluded with embolization microcoils. The patient then underwent hematoma clearing and experienced significant recovery of neurologic function after the second surgery, with the mJOA score reaching 13 points after 6 weeks.

## 3 Discussion

Intraoperative VAI occurs in cases of incorrect procedures entering the area occupied by a normal VA course or of correct procedures applied to a VA following an aberrant course.^[3–5]^ Patients are at the greatest risk when undergoing posterior instrumentation in upper cervical spine surgery.^[2]^ During cervical procedures adjacent to the VA course, sudden, rapid, and sometimes uncontrollable massive bleeding is the typical presentation of VAI. This typical presentation is impressive enough to establish a diagnosis of iatrogenic VAI, which is usually associated with a large amount of blood loss. A survey of large series demonstrated that blood loss ranges from 800 mL to >8500 mL (>3850 mL on average) when VAI occurs in an open field.^[1]^

In the present case, such noticeable bleeding did not occur. VAI probably occurred during the drilling of the posterior ring of the atlas according to photographs taken during surgery. Hemostasis was achieved by compression with only a few pieces of gelatin sponge, so the surgical team neglected the likelihood of a tiny rupture of the VA. To find the occult VAI in a timely manner, operators should be sensitive and pay sufficient attention to suspected bleeding at particular VAI-prone sites. Further examination might help to determine the potential risk.

**Figure 3:**
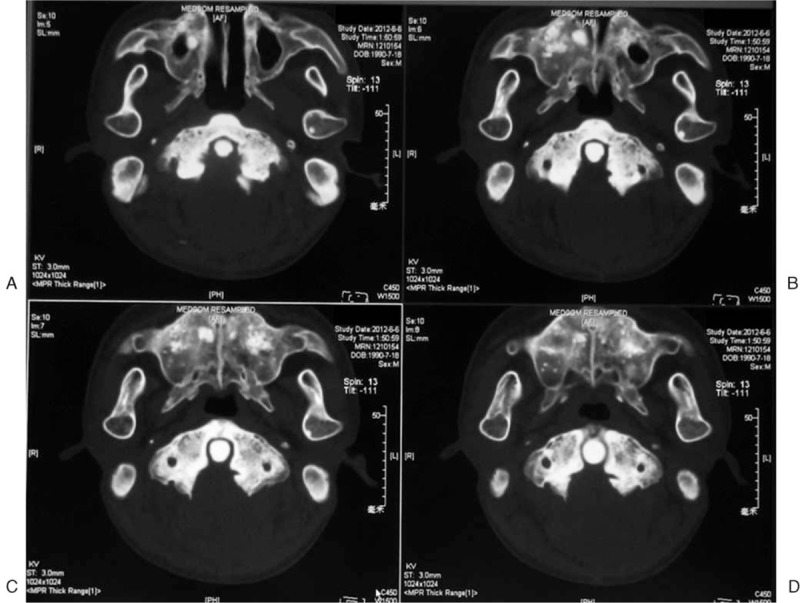
Continuous CT scans at the level of the atlas posterior arch. (A) The medial bone of the right VA groove was ground, whereas the left was preserved. (B–D) The bone groove ground during resection of the posterior ring was V-shaped, and the shallow grinding width was much larger than that deeper in the bone. VA = vertebral artery.

To our knowledge, this is the first case of an occult VAI presenting during an open cervical procedure in a patient with skeletal fluorosis. The morphological abnormality of bone may have contributed to the occurrence of iatrogenic VAI. In skeletal fluorosis with hypertrophy of the posterior ring and lamina, the operator tends to assume the lateral boundary of the spinal canal is wide, and thus, the grinding width is often larger than necessary. In the present case, as confirmed by CT, the groove was V-shaped (Fig. 3), and the shallow grinding width was much larger than that deeper in the bone to obtain a better vision and operation field during the operation. For C2 to C7, the widened groove in the shallow bone would not lead to problems as long as enough bone was reserved for instrumentation and fusion. However, when this groove was extended to C1, the expanded grinding procedure was more likely to injure the VA. Careful preoperative evaluation of the VA and identification of safe operation boundaries seem to be the key to preventing iatrogenic VAI, particularly in cases of morphological abnormality of bone or anomalous VA course.

## 4 Conclusion

VAI may occur during surgery without typical massive bleeding and lead to life-threatening late-onset pseudoaneurysm several weeks postoperatively. Surgeons should be sensitive and pay sufficient attention to suspected atypical bleeding at VAI-prone sites. The possibility of VAI should be considered when incision hematoma occurs after cervical surgery. In skeletal fluorosis, significant bone hypertrophy increases the risk of VAI in the upper cervical region. Compliance with the predetermined boundaries during the surgery will help to avoid VAI related to morphological abnormalities of the skeletal structure.
